# Evaluation of the Effect of a Cranberry Formulation in Reducing the Inflammatory State and Improving the Management of Symptoms in Patients with Symptomatic Uncomplicated Diverticular Disease: A Prospective, Open-Label, Single-Arm, Multi-Center, Pilot Study

**DOI:** 10.3390/pharmaceutics18010042

**Published:** 2025-12-28

**Authors:** Antonio Tursi, Stefano Rodinò, Ladislava Sebkova, Federica Furfaro, Silvio Danese

**Affiliations:** 1 Territorial Gastroenterology Service, ASL BAT, 76123 Andria, Italy; 2 Department of Medical and Surgical Sciences, School of Medicine, Catholic University, 00168 Rome, Italy; 3 Division of Gastroenterology, “Pugliese-Ciaccio” Hospital, 88100 Catanzaro, Italy; srodino@tin.it (S.R.); ladislavasebkova@seznam.cz (L.S.); 4 Division of Gastroenterology, IRCCS “San Raffaele” Hospital and University “Vita-Salute San Raffaele”, 20132 Milan, Italy; furfaro.federica@hsr.it (F.F.); sdanese@hotmail.com (S.D.)

**Keywords:** cranberry, DICA classification, fecal calprotectin, symptomatic uncomplicated diverticular disease

## Abstract

**Background/Objectives**: Low-grade inflammation and microbial imbalance have been thought to play a role in the pathogenesis of Symptomatic Uncomplicated Diverticular Disease (SUDD). We aimed to assess the efficacy of a cranberry formulation in reducing the inflammatory state of the colon and symptoms in SUDD patients. **Methods**: Twenty patients were enrolled in a prospective, multi-center, open-label, pilot study. We enrolled SUDD patients in whom fecal calprotectin (FC) was assessed at baseline and during the follow-up, with a baseline value ≥ 50 µg/g. Patients were treated with a gastroresistant formulation of cranberry (*Vaccinium macrocarpon*), one tablet/day for 4 weeks, followed by an 8-week observation period. The primary endpoint was to assess the efficacy of this gastroresistant cranberry formulation in reducing the inflammatory state of the colon by FC assessment. The secondary main endpoint was to assess the impact of this formulation on SUDD symptoms (assessed by the Visual Analog Scale, VAS). Intention-to Treat (ITT) and Per-Protocol (PP) analyses were performed. **Results**: At baseline, the mean FC value was 110 ± 118 μg/g; it was 72 ± 24 μg/g and 82 ± 19 μg/g after 4 weeks of treatment, and after a further 8 weeks of observation, it was significantly reduced on both ITT (*p* = 0.0001) and PP (*p* = 0.001). About the secondary main endpoint (namely symptoms of SUDD), the mean values according to the VAS were reduced significantly both at the end of the treatment and after 8 weeks post treatment. **Conclusions**: This gastroresistant formulation of cranberry may be able to reduce inflammation and symptoms in SUDD patients. Furthermore, large studies have to confirm these preliminary and promising results.

## 1. Introduction

The diverticula are extro-flexions of the colonic wall, which are formed in the “*loci minoris resistentiae*” of the colon, and they generally correspond to the point where the vasa recta perpendicularly cross the colonic wall.

People with colonic diverticulosis are generally asymptomatic during their lifetime, and approximately 20% of them develop the so-called “Diverticular Disease” (DD), which includes all clinical manifestations linked to diverticula (bleeding, diverticulitis, segmental colitis, or a particular condition defined as symptomatic uncomplicated diverticular disease (SUDD)) [1]. SUDD has a significant impact on the quality of life of patients as it causes abdominal pain, bloating, and alteration in bowel movements, even if without signs of inflammation or evident anatomic lesions [2].

The patho-physiological mechanisms leading to diverticula formation are not yet completely clear, but age, diet, anatomic factors, genetic predisposition, intestinal motility, and inflammation certainly play a key role in their genesis. In particular, recent studies have shown that alterations in the intestinal microbiota and the occurrence of microscopic inflammation are two important risk factors for the development of DD [3,4].

Currently, there are no clear guidelines for the treatment of SUDD. The recommended management includes a diet rich in fruits and vegetables, in addition to the administration of rifaximin; mesalamine has also been shown to be effective in the management of symptoms associated with SUDD, while for probiotic use, there is promising but insufficient evidence on their efficacy [2,5,6,7].

Cranberries are a group of evergreen dwarf shrubs or trailing vines in the subgenus *Oxycoccus* of the genus *Vaccinium*. In the United Kingdom, cranberry may refer to the native species *Vaccinium oxycoccos* [8], while in North America, cranberry may refer to *Vaccinium macrocarpon* [9]. *Vaccinium oxycoccos* is cultivated in central and northern Europe, while *Vaccinium macrocarpon* is cultivated throughout the Northern United States, Canada, and Chile.

Cranberries have attracted the attention of clinicians because they seem to be effective in reducing bacterial overgrowth and bacterial adhesion to the epithelium. In particular, their activity seems to be related to the presence of proanthocyanidins (PAC), flavonoids, and hydroxycinnamic acids. For example, cranberries have been shown to reduce the adhesion of bacteria such as *E. Coli* at the urinary tract level and may decrease the recurrence of urinary tract infections [10,11]. Further studies on cranberries have shown that they are also effective against other microorganisms such as *Helicobacter pylori*, *Streptococcus mutans*, *Porphyromonas gingivalis*, *Staphylococcus aureus*, *Pseudomonas aeruginosa*, *Cryptococcus neoformans*, *Haemophilus influenzae*, *Candida albicans*, and extraintestinal pathogenic *Escherichia coli* [12,13,14,15,16,17,18,19,20,21,22]. Finally, new studies have shown there is a correlation between cranberry and gut microbiota. Cranberries reduce the adhesion capacity of microorganisms, have an anti-inflammatory effect, and reduce the production of biofilm [23].

In SUDD patients, there is a strong relationship between gut microbiota imbalance and the overexpression of pro-inflammatory taxa, severity of symptoms, low-grade inflammation, and endoscopic severity of the disease [24,25]. Thus, thanks to these characteristics, cranberries could play a role in the management of SUDD symptoms and in reducing intestinal inflammation in these patients.

We aimed, therefore, to assess whether cranberry intake in a gastroresistant formulation, which allows the release of active components directly at the intestinal level, can be effective in patients with SUDD in reducing the inflammatory state, also improving the management of its typical symptoms, such as abdominal pain, bloating, and changes in bowel movements.

## 2. Materials and Methods

This was a prospective, open-label, single-arm, multi-center, pilot study (conducted at Territorial Gastroenterology Service, ASL BAT, Andria (BT), Italy; Division of Gastroenterology, “Pugliese-Ciaccio” Hospital, Catanzaro, Italy; Division of Gastroenterology, IRCCS “San Raffaele” Hospital and University “Vita-Salute San Raffaele”, Milan, Italy). It was designed to assess whether this formulation is suitable for further, larger, and controlled studies.

The study consists of one screening visit, a 4-week treatment period and an 8-week follow-up, for a total of 12 weeks from baseline per completed patient. It was sponsored by AlfaSigma S.p.A. (Bologna, Italy), which also provided some kits and devices for the study (see below the paragraph *Experimental Design*).

This clinical study was designed in accordance with the ICH Harmonized Tripartite Guidelines for Good Clinical Practice, with applicable local regulations (including European Directive 2001/20/EC, US Code of Federal Regulations Title 21, and the Japanese Ministry of Health, Labor, and Welfare), and with the ethical principles laid down in the Declaration of Helsinki.

The study protocol (PSC-DS PACS 18) was approved by the Ethics Committee of the Humanitas Research Hospital (Rozzano, Milan, Italy) on 18 October 2018, and by each center involved in the study. In Italy, prospective observational studies are regulated by the guidelines of the Italian Medicines Agency (AIFA, Agenzia Italiana per il Farmaco). Until 1 January 2023, prospective studies only required authorization from the ethics committee, while from that date the guidelines require registration in the AIFA Register of Observational Studies (RSO). This registry was activated on 31 January 2023; since our study was approved in October 2018, well before the new rules came into force, our study did not need to be registered. This information is readily available by consulting the institutional website https://www.aifa.gov.it/registro-studi-osservazionali (Accessed on 18 December 2025).

### 2.1. Patients Population

Twenty patients suffering from SUDD agreed to give written informed consent and were enrolled in a competitive fashion. The diagnosis of SUDD was assessed according to well-defined criteria (moderate-to-severe left-lower quadrant pain lasting ≥ 24 h in patients with diverticulosis, without any sign of acute diverticulitis) [1].

### 2.2. Inclusion Criteria

Patients were included in the study according to the following criteria:

➢ Age ≥ 18 years;
➢ Diverticulosis diagnosed by colonoscopy and scored according to the DICA classification [26] (only DICA 1 patients were enrolled);
➢ SUDD diagnosis, according to the well-defined criteria [1];
➢ Positivity for fecal calprotectin (FC). We used a kit provided by SOFAR S.p.A. (Trezzano Rosa, Italy), and the cut-off level for FC positivity was ≥50 µg/g;
➢ Ability to provide written informed consent.

### 2.3. Exclusion Criteria

Patients were excluded from this study if they met any of the following criteria:

➢ Diagnosis of Irritable Bowel Syndrome (IBS) according to the Rome IV criteria [27];
➢ Patients with a current or past medical history of being positive for acute diverticulitis (defined as the acute inflammation of diverticula with thickening of the colonic wall, fat stranding, and with or without complications such as abscesses and/or stenosis and/or fistulas) [1];
➢ Patients with moderate-to-severe DD detected at endoscopy (namely, DICA 2 and DICA 3 patients) [26];
➢ Intake of antibiotics, mesalamine, probiotics, and Vitamin K antagonists (e.g., Warfarin) in the 30 days prior to screening;
➢ Presence of any relevant organic, systemic, or metabolic disease (particularly a significant history of cardiac, renal, neurological, psychiatric, oncology, endocrinology, metabolic, or hepatic disease), or abnormal laboratory values that were deemed clinically significant in the investigator’s opinion on the basis of the normal predefined ranges;
➢ Ascertained intestinal organic diseases different from DD, including celiac disease, food allergies, or inflammatory bowel diseases (Crohn’s disease, ulcerative colitis, infectious colitis, ischemic colitis, and microscopic colitis);
➢ Active malignant neoplasm of any type, or a history of malignancy (patients with a history of other malignancies that have been surgically removed and who have no evidence of recurrence for at least five years before study enrollment are also acceptable);
➢ Pregnant females or females of childbearing potential without effective contraceptive methods;
➢ Inability to conform to the protocol;
➢ Treatment with any investigational drug within the previous 30 days;
➢ Recent history or suspicion of alcohol abuse or drug addiction;
➢ Inability to sign the informed consent.

### 2.4. Study Treatment

The investigational product in this study is a gastroresistant formulation of cranberry (*Vaccinium macrocarpon Aiton*; source: Urophenol, product code: VD00240004; DIANA FOOD CANADA Inc. (Champlain, Québec, QC, Canada), and is to be administered as 1 tablet per day, swallowed with liquids, for 4 weeks.

Each tablet contains cranberry extract titled 15% proanthocyanidins (PACS) 240 mg as its active ingredient, together with other components (cellulose 304.4 mg, mannitol 15 mg, silicon dioxide 5.8 mg, reticulated sodium carboxymethylcellulose 9 mg, and magnesium salts of fatty acids 5.8 mg). Finally, the tablet is coated with Eudragard^®^ biotic E1207 (Evonik Corporation, Rellinghauser Strasse 1—11, 45128 Essen, Germany) 20.4 mg, triethyl citrate E1505 1 mg, and talc 10.2 mg in order to guarantee gastroresistance and dissolution through the colon. This allows the product to reach the right intestinal district where it can act, since we are talking about a disease of the colon.

In accordance with the ICH Good Clinical Practice guidelines for clinical studies’ conduction, any surplus treatment and all packages should be returned to the Sponsor, even in the case of withdrawal or discontinuation of the study.

Patient treatment compliance should be, at a minimum, at 80% and no more than 120%.

In the case of poorly controlled symptoms, patients were allowed to take paracetamol 1000 mg (up to three times per day) for abdominal pain (Visual Analogic Scale, VAS, >6).

### 2.5. Primary Endpoint

The primary endpoint of the study was the reduction in the intestinal inflammatory state in patients with SUDD, assessed by measuring FC levels at each visit from baseline to the follow-up visit.

### 2.6. Secondary Endpoints

The secondary endpoints of the study were considered as follows:

➢ The reduction in typical symptoms of SUDD (abdominal pain, bloating, diarrhea, and constipation), evaluated through the VAS;
➢ Daily stool consistency and frequency, evaluated from the baseline by the Bristol Stool Chart [28] through patient diaries for the entire duration of the study (treatment period and follow-up);
➢ Reduction in the serum inflammatory state, evaluated by measuring serum C-reactive protein levels at the end of treatment and at the follow-up visits versus the baseline value;
➢ Overall patient satisfaction with the treatment, assessed by the VAS, collected at the end of the study (week 12).

### 2.7. Experimental Design

As stated, this was an interventional, prospective, open-label, single-arm, pilot study.

**Figure 1:**
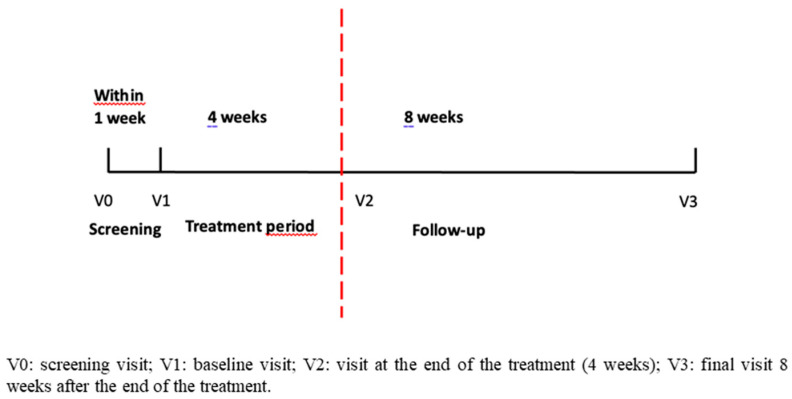
Schematic representation of the experimental design.

The study consisted of a screening visit, a 4-week treatment period, and an 8-week follow-up, for a total of 12 weeks per completed patient. The screening visit may occur up to 7 days prior to the baseline visit. Laboratory reports for blood tests (hematology, chemistry blood tests, and serum C-reactive protein dosage) were performed at entry in the Hospital Local Laboratory, while urine pregnancy tests were performed on site with dipsticks supplied by the sponsor. All tests had to be available before the baseline visit (V1) (Figure 1).

FC assessment was performed at the investigational site with a diagnostic device and disposable material supplied by the sponsor.

The screening visit included the following: obtaining informed consent before any study-specific procedures; collection of patient demographics; determining if the patient meets the inclusion/exclusion criteria; reviewing of the prior and concomitant medication history; vital signs; urine pregnancy test; physical examination; confirmation of the SUDD with diverticulosis according to the DICA1 score [24]; exclusion of acute diverticular inflammation according to a CT scan; blood sampling (see flow chart); providing a stool kit for V1; and dispensing of patient diary.

The baseline visit (treatment start, V1) included the following: confirmation whether the patient meets the inclusion/exclusion criteria; reviewing of any new concomitant medication; vital signs; physical examination; dispensing the study product; patient diary check (Bristol scale and diet details); dispensing of patient diary; stool sample collection; providing a stool kit for V2; assessment of adverse events; symptom evaluation (abdominal pain, bloating, diarrhea, and constipation) by the VAS; and FC assessment.

The end of the treatment visit (end of the treatment, week 4, V2) included the following: vital signs; physical examination; review of concomitant medications; assessment of adverse events; blood sampling; stool sample collection; patient diary check (Bristol scale and diet details); dispensing of patient diary; accountability of the study product and retrieval; providing a stool kit for V3; symptom evaluation (abdominal pain, bloating, diarrhea, and constipation) by the VAS; and FC assessment.

The final visit (V3) (follow-up, 8 weeks after the treatment period, week 12, V3) included the following: vital signs; physical examination; blood sampling; stool sample collection; FC assessment; symptom evaluation (abdominal pain, bloating, diarrhea, and constipation) by the VAS; patient diary check (Bristol scale and diet details); patient diary collection; review of concomitant medications; assessment of adverse events; and performing the VAS to assess overall patient satisfaction with the study treatment.

### 2.8. Safety Evaluation

Adverse events were continuously monitored during the study and defined as the appearance of undesirable sign(s), symptom(s) or medical condition(s) that occur after patient signed informed consent had been obtained. Abnormal laboratory values occurring after the informed consent signature process constituted adverse events only if they induced clinical signs or symptoms, were considered clinically significant, or required therapy or changes in the study medications. In each case, the investigator must decide whether the change in a laboratory parameter was clinically relevant and therefore representative of an adverse event according to the above criteria and the clinical state of the patient.

No particular side effects were found in relation to the study product. However, if taken in quantities greater than that recommended, cranberry could induce laxative effects. In any case, this side effect is transitory.

### 2.9. Lifestyle and Eating Habits to Adopt During the Study

Patients were asked to complete a food diary of the quantity that they ate every day.

They were provided with some advice about the eating and lifestyle habits to follow during the study, as follows:

#### 2.9.1. General Recommendations

- Take plenty of liquids (water, at least 1.5–2 L per day, but also consuming broth or herb teas);
- Exercise regularly (at least 20–30 min a day);
- Avoid cigarette smoking;
- Avoid swallowing food quickly and try to chew slowly.

#### 2.9.2. Food to Eat in Moderation

- Alcoholic beverages;
- Spicy spices (such as pepper and chili);
- Chocolate, snacks, or sweets;
- Sausages.

### 2.10. Treatment Compliance

The patients were treated with the study product, 1 tablet/day, swallowed with liquids, for 4 weeks and returned all unused study treatments, including empty packages, to the site. The product accountability/compliance was performed by the investigator, by performing product accountability, and checked by the monitor during monitoring visits.

In accordance with the ICH Good Clinical Practice guidelines for clinical studies’ conduction, any surplus treatment and all packages were returned to the sponsor, even in the case of withdrawal or discontinuation of the study.

Patient treatment compliance was, at a minimum, at 80% and no more than 120%.

### 2.11. Statistical Analysis

Data were analyzed using SAS software version 9.4.

Due to the pilot nature of the study, no formal hypotheses have been pre-specified, so no hypothesis testing was performed. Descriptive statistics were provided in order to explore and describe the treatment’s effects and its safety profile.

Continuous variables were presented as the mean values ± standard deviation (SD), median value with interquartile range, minimum, and maximum; categorical variables were summarized as counts and percentages. Chi-square testing for paired data was applied, and Confidence Intervals (CI) at a 95% level were provided.

The following study populations were considered:

- Full Analysis Set (FAS): this set consists of all patients;
- Safety Set: this set consists of all patients who receive at least one dose of study treatment;
- Per Protocol Set (PPS): this set consists of all patients who complete the study without any significant protocol violation;
- Intention-to-Treat Set (ITT): this set consists of all patients who receive at least one dose of study treatments and have at least one post-baseline efficacy assessment.

## 3. Results

### 3.1. Baseline Characteristics

Twenty-two patients were screened in the study, and all of them were included in the FAS. In SAS, twenty patients (90.9%) were included, and two patients (9.1%) were excluded because of a screening failure (FC < 50 µg/g), and they did not take any dose of the product. Regarding the ITT Analysis Set, nineteen patients were analyzed because, besides the two patients mentioned above, one patient was excluded due to loss to follow-up and failing to undergo post-baseline assessment. Moreover, regarding the PPS, nine patients (40.9%) were included due to the following reasons: two patients (9.1%) were excluded because there were screening failures, and eleven patients (50.0%) were excluded because of a major protocol deviation during the study (three patients took drugs prohibited during the study: one took nimesulide, one azithromycin, and the last one ibuprofen; three patients delayed their control visit due to concomitant COVID-19; five patients delayed completing the documents).

**Figure 2:**
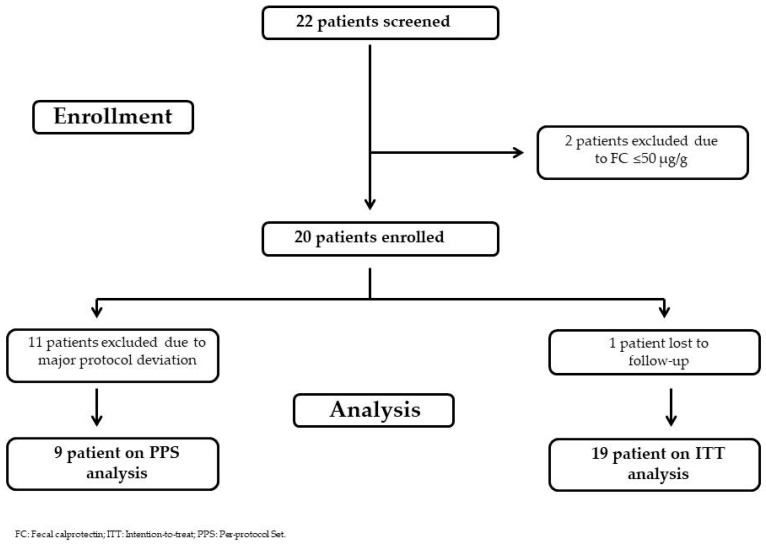
Flow-chart of the study.

The final flow-chart is reported in Figure 2.

### 3.2. Demographic and Other Baseline Characteristics

Regarding the demographics and baseline characteristics, all information are presented in Table 1.

The mean age was 58 ± 9 years, with a minimum value of 44 years and a maximum value of 74 years. Five patients (26.3) were female and all patients were Caucasian. Only one female patient performed a urine pregnancy test with a negative result; the other women did not perform the test due to their menopause status.

All patients received one package with 45 tablets, and all patients registered a compliance of 100% during the study.

### 3.3. Primary Endpoint

The mean values of FC expression at V1, V2, and V3 were 110 ± 118 μg/g, 72 ± 24 μg/g, and 42 ± 28 μg/g, with a significant drop during follow-up (<0.0001).

At V1, all enrolled patients (100.0%) had a value of fecal calprotectin ≥ 50 μg/g, indicating the presence of an inflammatory state. The mean value was 110 ± 118 μg/g, with a minimum value of 53 μg/g and a maximum value of 591 μg/g (IQR 72–99 μg/g).

At V2, only one patient (5.3%) failed to undergo the assessment. Five patients (27.8%) had a value of fecal calprotectin ≥ 50 μg/g; among the five patients with a value of fecal calprotectin ≥ 50 μg/g, the mean value was 72 ± 24 μg/g at V2, with a minimum of 52 μg/g and a maximum of 112 μg/g. Finally, 13 patients (72.2%) had a value < 50 μg/g, indicating the absence of an inflammatory status.

At V3, fifteen patients (83.3%) had a value < 50 μg/g, whereas three patients (16.7%) had a value ≥ 50 μg/g (65 μg/g, 82 μg/g, and 84 μg/g, respectively).

**Figure 3:**
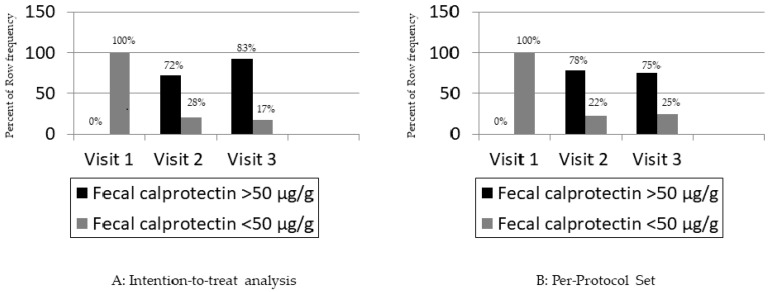
Primary endpoint of the study: fecal calprotectin expression at baseline and during the follow-up.

A Chi-square test for paired data was applied to evaluate whether the transition from the inflammatory state to the non-inflammatory state over time could be considered statistically significant. The ITT *p*-value was <0.0001, indicating that the patients’ inflammatory condition improved during the entire study duration (Figure 3A). Repeating the analysis on the PPS, the result did not change; the *p*-value of the Chi-square test per paired data was 0.001, indicating a statistically significant transition (Figure 3B).

### 3.4. Secondary Endpoints

#### 3.4.1. Reduction in Typical Symptoms of SUDD

At the end of each study week, patients registered the intensity of typical SUDD symptoms (abdominal pain, bloating, diarrhea, and constipation) in the patients’ diaries using a VAS.

The mean baseline values (V1) were as follows:

- Abdominal pain: 7.2 ± 2.7;
- Diarrhea: 3.6 ± 3.6;
- Constipation: 5.9 ± 2.9;
- Bloating: 8.0 ± 2.0.

At the end of the treatment (week 4, V2), the mean values were changed as follows:

- Abdominal pain: 1.4 ± 3.3 (*p*-value = 0.0005);
- Diarrhea: 0.1 ± 0.3 (*p*-value from paired t test = 0.0075);
- Constipation: 0.9 ± 2.0 (*p*-value < 0.0001);
- Bloating: 1.8 ± 3.0 (*p*-value = 0.0001);

Finally, at the end of the study (week 8 after ending treatment, V3), the mean values were changed as follows:

- Abdominal pain: 3.6 ± 4.0 (*p*-value = 0.036);
- Diarrhea: 1.5 ± 2.7 (*p*-value = 0.29);
- Constipation: 2.2 ± 3.2 (*p*-value = 0.0016);
- Bloating: 3.5 ± 3.9 (*p*-value = 0.0034).

#### 3.4.2. Daily Stool Consistency and Frequency

During the study, the patients recorded the number of evacuations and their type according to the Bristol scale daily. The mean Bristol score per week was 4 during the follow-up, as follows:

- During the first week, the most frequent Bristol type was type 4 (37.6%); during week 2, week 3, and week 4, the most frequent stool type was type 4 (29.3% during week 2, 34.6% during week 3, and 27.8% during week 4);
- Type 4 was also the most frequent Bristol type during week 5 (26.1%), during week 6 (31.4%), during week 7 (30.3%), and during week 8 (23.9%).

#### 3.4.3. Reduction in Serum Inflammatory State

The reduction in the serum inflammatory state was evaluated measuring serum C-reactive protein levels at V0, V2 and V3.

At V0, the mean value was 3.44 ± 1.17 mg/L, with a minimum value of 3.11 mg/L and a maximum value of 8.26 mg/L; at V2, the mean value was 3.56 ± 1.19 mg/L, with a minimum value of 3.13 mg/L and a maximum value of 7.52 mg/L; at V3, the mean value was 3.98 ± 2.03 mg/L, with a minimum value of 3.14 mg/L and a maximum value of 10.3 mg/L.

To evaluate whether the change in C-reactive protein was statistically significant with respect to the baseline value (V0), a Wilcoxon test for paired data was applied at V2 and V3 due to the violation of the normality check distribution applying the Kolmogorov–Smirnov test (*p*-value < 0.01). On both study visits, the change could not be considered statistically significant because the *p*-value was 0.70 at V2 and 0.14 at V3.

#### 3.4.4. Overall Patient Satisfaction with Treatment

At the end of the study (V3), the overall patient satisfaction with the treatment was evaluated.

Of the 15 patients included in the ITT Analysis Set who completed the visit and the satisfaction evaluation with the VAS, the mean value was 7.73 ± 1.28, with a minimum value of 6.00 and a maximum value of 9.90.

### 3.5. Safety

No adverse events were recorded during the study period.

## 4. Discussion

Although the epidemiological data show clearly that DD is a disease with a significant impact worldwide [29,30,31,32], its treatment is still under debate. This is probably due to the incomplete knowledge of its pathogenesis, which leads to several hypotheses. The occurrence of low-grade inflammation [1], together with gut microbiota [24,25] and oxidative imbalance [33], are some of them, and several therapeutic approaches, ranging from non-absorbable antibiotic rifaximin to mesalamine, have been proposed to treat SUDD [34].

A new approach to SUDD is represented by nutraceuticals. These are products such as meals or nourishment supplements that produce medical or fitness advantages, inclusive of the prevention/treatment of any type of sickness [35]. These formulations, such as the combination of cranberry with other nutrients (*Boswellia serrata*, quercitin, and inulin) [36], *Curcuma longa* with *Boswellia serrata* [37], or *Hericium erinaceus* [38], have been recently found effective in treating symptoms in SUDD patients.

Cranberries are rich in a variety of biologically active components, such as polyphenols (proanthocyanidins, chlorogenic acid, flavonols, anthocyanins, caffeic acid, etc.), triterpenoids, and other nutrients. The current literature has shown that the chemical components extracted from cranberry fruit have a lot of pharmacological effects, such as antioxidant, anti-inflammatory, and anti-cancer effects [39]; they are commonly used clinically in the treatment of cardiovascular diseases, lowering blood pressure, reducing the adhesion of bacteria such as *E. Coli* at the urinary tract level, and therefore reducing the recurrence of urinary tract infections and inflammation [10,11].

No previous studies have investigated cranberry as a single agent in managing SUDD patients. According to its biological characteristics [10,11,39], and its extremely favorable safety profile [40], this nutraceutical product could be promising in treating SUDD patients. This prospective, pilot study assessed for the first time the impact of cranberry on the colonic inflammation in SUDD patients according to FC expression. We found for the first time that a gastroresistant cranberry formulation is able to reduce colonic inflammation, reducing significantly FC expression in these patients. This result was obtained both on the ITT set and on the PPS; this means that, even excluding the confounding factors, cranberry seems to be able to reduce FC expression in SUDD patients, reducing therefore the inflammation. This formulation was also able to control symptoms in SUDD, which were all significantly reduced. This reinforces the hypothesis that SUDD symptoms may be mainly linked to low-grade inflammation, which could be sustained by other factors (such as gut microbiota imbalance) [41].

Cranberry seems therefore to work as *Hericium erinaceus*, a medicinal fungus that recently showed efficacy in the same patient setting [38]. However, the use of this gastroresistant cranberry formulation could be more useful due to another interesting finding. We found that FC expression and symptoms were significantly lower than baseline also during the 8-week observational period after stopping the treatment. This means that this product could be administered as cyclic treatment, reducing the costs for the patients and improving long-term compliance with therapy.

The last interesting finding of this study is that this gastroresistant cranberry formulation is very well tolerated, since no adverse events were recorded. We confirm therefore that, in general, cranberry is very safe [42], and it can also be used in patients with a high risk of adverse events owing to physiology, multimorbidity, and polypharmacy.

Of course, this study has some limitations. The first is that this is a pilot study conducted in a small sample population. However, this is a correct approach in assessing the characteristics of a new product, and further controlled studies (e.g., with a placebo arm) with larger populations can be planned according to the results of this pilot study. Another limit of this type of study is that confounding factors cannot be easily and clearly controlled, and this could limit the results. About the results, the PPS results were obtained in a small number of patients, and this could be a limitation. However, we obtained the same results both on the ITT set and the PPS, limiting therefore the impact of a small population on the PPS analysis. Finally, the selection of a DICA 1 population could be a limitation. The DICA 1 population represents the majority of people with diverticulosis detected during colonoscopy [26] and, in terms of symptoms, can be considered as a mild disease [43]. On the contrary, DICA 2 and DICA 3 patients represent moderate-to-severe DD, with diverticular inflammation in the large majority of cases, and are more often associated with acute diverticulitis [43]. We planned to select only DICA 1 patients because previous studies found nutraceutical formulations in DICA 2 patients ineffective when used as a single treatment [36,37]. However, this does not mean that nutraceuticals cannot also be used in DICA 2 and DICA 3 populations, but it means that nutraceuticals can be advised only as adjunctive therapy in these populations.

## 5. Conclusions

In conclusion, this pilot study found that a gastroresistant formulation of cranberry may be able to reduce inflammation and symptoms in SUDD patients. Further studies are needed to confirm these preliminary and promising results. In particular, larger studies expanding the sample size and adopting a more robust study design, together with prospecting registration in a recognized clinical trial registry, are advisable for enhancing the results of future research in this setting.

## Figures and Tables

**Table 1:** Baseline and demographic characteristics.

**Overall**	**(N = 19)**
**Age (years)**	
Mean ± SD	58 ± 9
Median	58
IQR	50–63
Min.–max	44–74
**Gender**	
Female n. (%)	5 (26.3)
Male n. (%)	14 (73.7)
**Ethnic group**	
Caucasian n. (%)	19 (100.0)
**Any medical history recorded?**	
**Yes n. (%)**	9 (47.4)
**No n. (%)**	10 (52.6)
**Medical records**	**(N = 19)**
Appendectomy n. (%)	1 (5.6)
Arterial hypertension n. (%)	1 (5.6)
Benign prostatic hyperplasia n. (%)	2 (11.1)
Blood hypertension n. (%)	5 (27.8)
Cesarian section n. (%)	1 (5.6)
Cholecistectomy n. (%)	3 (16.7)
Essential tremor n. (%)	1 (5.6)
Gastro-esophageal reflux disease n. (%)	1 (5.6)
Hashimoto thyroiditis n. (%)	1 (5.6)
OSAS n. (%)	1 (5.6)
Depressive syndrome n. (%)	1 (5.6)

## Data Availability

The data can be made available from the sponsor upon reasonable request.

## Abbreviations

The following abbreviations are used in this manuscript:

SUDD	Symptomatic Uncomplicated Diverticular Disease
ITT	Intention-to-Treat
PP	Per-Protocol
VAS	Visual Analogic Scale
